# Supplementary figures and images for: Assessment of DNA/RNA Defend Pro: An Inactivating Sample Collection Buffer for Enhanced Stability, Extraction-Free PCR, and Rapid Antigen Testing of Nasopharyngeal Swab Samples
Source: Int J Mol Sci. 2024 Aug 22;25(16):9097. doi: 10.3390/ijms25169097 (PMC11354787; doi:10.3390/ijms25169097)

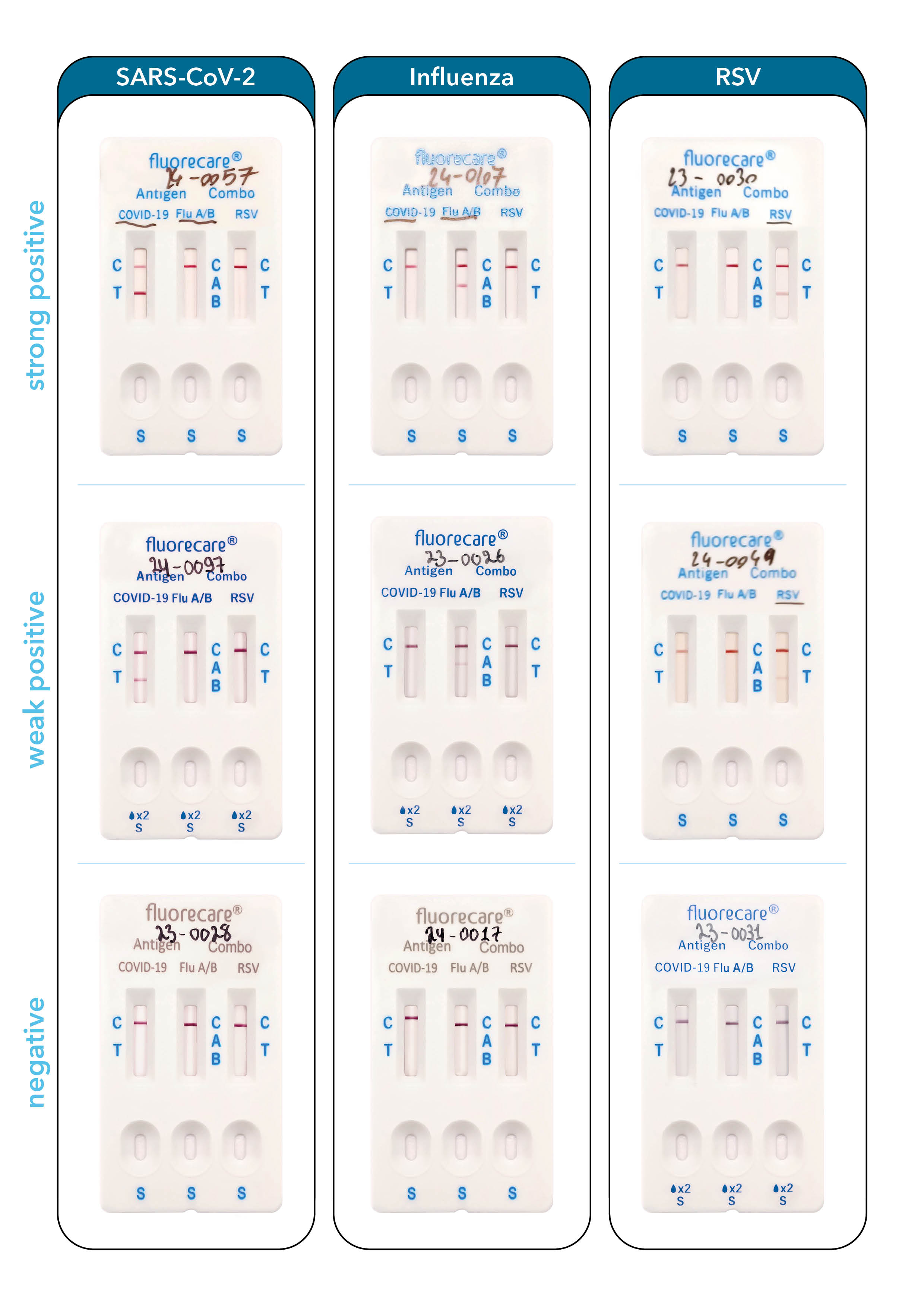

Supplement: Supplementary file 1 [file ijms-25-09097-s001.zip › Figure S1.jpg]
